# Alcohol and the Human Body

**Published:** 1908-03

**Authors:** 


**Alcohol and the Human Body.** By Sir VICTOR HORSLEY and MARY D. STURGE. Pp. xxv., 370. London: Macmillau and Co. Limited. 1907.

This is a small book which is intended not only for the use of the medical profession, but also for the general public, for whom a glossary of medical terms is inserted at the end of the work. Being concise in its arrangement, it gives a large amount of information, and covers a very wide field in a relatively small space. The effect of alcohol on most of the organs of the body is considered in some detail, and a great deal of care has been expended in obtaining the most reliable information with regard to the statistics. The statements and opinions of experts are freely quoted throughout. Chapters on the special effect of alcohol on children, and on the influence of prenatal alcoholism upon the race are included, while Dr. Newsholme adds an interesting chapter on the influence of drinking on the national health. The work contains a large number of illustrations, some of which—notably those on the nervous system, which have been prepared from sections supplied by Dr. F. W. Mott—are particularly good. We can confidently recommend it to all who are interested in the harmful effects wrought by alcohol on the individual and on the race.

